# On solving the chlorine transport model via Laplace transform

**DOI:** 10.1038/s41598-022-14655-3

**Published:** 2022-07-15

**Authors:** A. F. Aljohani, A. Ebaid, E. A. Algehyne, Y. M. Mahrous, P. Agarwal, M. Areshi, H. K. Al-Jeaid

**Affiliations:** 1 Computational & Analytical Mathematics and Their Applications Research Group, Department of Mathematics, Faculty of Science, University of Tabuk, Tabuk, 71491 Saudi Arabia; 2 Department of Studies and Basic Sciences Faculty of Community, University of Tabuk, Tabuk, Saudi Arabia; 3 Department of Mathematics, Anand International College of Engineering, Jaipur, 303012 India; 4 Department of Mathematics, Harish-Chandra Research Institute, Allahabad, 211 019 India; 5 International Center for Basic and Applied Sciences, Jaipur, 302029 India; 6 Department of Mathematical sciences, Umm Al-Qura University, Makkah, Saudi Arabia

**Keywords:** Engineering, Applied mathematics

## Abstract

This paper analyzes the two-dimensional chlorine-transport model in pipes. The studied model is in the form of a second-order partial differential equation with a set of boundary conditions. Obtaining exact solution for the current model is a challenge due to the nature of the involved boundary conditions, especially, when applying the Laplace transform. However, such difficulties are solved via implementing the method of residues. The exact solution is obtained in terms of the Bessel functions. The expression for a dimensionless cup-mixing average concentration is also derived analytically. The proposed approach is validated via numerical examples for comparing the results with those in the literature. The present analysis/approach is effective/straightforward and can be further applied on other similar models under different boundary conditions.

## Introduction

The study of the water quality is a growing field of research due to its importance in industry and engineering sciences. As a result of its importance in the quality of drinking water, chlorine is used in most parts of the world as a guarantee for the distribution of safe drinking water^1,2^. To ensure the quality of the water, part of the chlorine must remain to prevent the survival and growth of any microorganisms that may be present during the movement of water in the networks. Therefore, maintaining a certain level of chlorine ensures that no harmful by-products are formed within the distribution networks. In Ref.^3^, the percentage of chlorine concentration that ensures the avoidance of public health risks has been determined as 0.2 mg l$^{-1}$. Accordingly, the successful management of ensuring the quality of drinking water requires adherence to the aforementioned chlorine concentration and ensuring that this percentage does not rise above the specified limit. The basic model of chlorine transport was established by Biswas et al.^4^ in the form:

1 $$\begin{aligned} \frac{\partial u}{\partial x}=\frac{A_0}{r}\frac{\partial }{\partial r} \left( \frac{1}{r}\frac{\partial u}{\partial r}\right)-A_1u, \end{aligned}$$

and

2 $$\begin{aligned}&u(0,r)=1,\qquad \qquad \qquad \qquad 0\le r\le 1, \end{aligned}$$

3 $$\begin{aligned}&\frac{\partial }{\partial r}u(x,0)=0, \qquad \qquad \qquad \quad 0\le x\le 1, \end{aligned}$$

4 $$\begin{aligned}&\frac{\partial }{\partial r}u(x,1)+A_2u(x,1)=0, \qquad 0\le x\le 1, \end{aligned}$$

where *u*(*x*, *r*) is the chlorine concentration, and $A_0$, $A_1$ and $A_2$ are dimensionless parameters related to the chlorine decay. Further, details about the dimensionless parameters and derivation of the Eqs. (1)–(4) can be found in Ref.^4^. The purpose of the present work is to solve the system (1)–(4) through applying the Laplace transform (LT). The LT is a well-known and effective approach to solving various scientific models in physics and engineering^5–10^. Ebaid and Al sharif^6^ applied the LT on the ODE governing the heat transfer of nanofluids suspended with carbon-nanotubes. Ebaid et. al^7^ provided the analytic solution for a class of singular boundary value problems (SBVPs) via the LT. Khaled^8^ obtained the exact solution of the model describing the radiation effect on MHD Marangoni convection over a flat plate. Ebaid et. al^9^ solved a general class of SBVPs with applications in nanofulids via the LT. Bakodah and Ebaid^10^ addressed the Ambartsumian delay equation using the LT. A variety of other of LT applications in addition to other transforms can be found in Refs.^11–30^. In this paper we consider the application of the LT for the solution of the system (1)–(4). The paper is structured as follows. The LT approach is applied in “Application of LT” on Eqs. (1)–(4). Section “Analysis and exact solution of the chlorine decay model” is devoted to obtaining exact solution. Section “Discussion of results” analyses the results and discusses their physical meaning. In addition, the results are to be compared with those in Ref.^4^. Finally, Section “Conclusion” outlines the main conclusions.

## Application of LT

Applying LT on Eq. (1) with respect to the variable *x*, we can write

5 $$\begin{aligned} L\left( \frac{\partial u(x,r)}{\partial x} \right)=L\left( \frac{A_0}{r}\frac{\partial }{\partial r} \left( \frac{1}{r}\frac{\partial u(x,r)}{\partial r}\right) \right)-L\left(A_1u(x,r)\right), \end{aligned}$$

which gives

6 $$\begin{aligned} sU(s,r)-u(0,r)=\frac{A_0}{r}\frac{\partial }{\partial r} \left( \frac{1}{r}\frac{\partial U(s,r)}{\partial r}\right)-A_1U(s,r), \end{aligned}$$

where $L(\cdot )$ and *s* denote the Laplace operator and variable, respectively. After introducing the BC given in (2), Eq. (6) becomes

7 $$\begin{aligned} \left(s+A_1\right)U(s,r)-1=\frac{A_0}{r}\frac{d }{d r} \left( \frac{1}{r}\frac{d U(s,r)}{d r}\right). \end{aligned}$$

Re-arranging Eq. (7), yields

8 $$\begin{aligned} \frac{d^2U(s,r) }{d r^2}+ \frac{1}{r}\frac{d U(s,r)}{d r}-\left( \frac{s+A_1}{A_0} \right)U(s,r)=-\frac{1}{A_0}, \end{aligned}$$

which is the well known Bessel differential equation with inhomogeneous part $(-\frac{1}{A_0})$. The solution of Eq. (8) is given by

9 $$\begin{aligned} U(s,r)=c_1J_0\left(i\sqrt{ \frac{s+A_1}{A_0} }r\right) +c_2Y_0\left(i\sqrt{ \frac{s+A_1}{A_0} }r\right)+\frac{1}{s+A_1}, \end{aligned}$$

where $J_0\left(\cdot \right)$ and $Y_0\left(\cdot \right)$ are Bessel functions and $c_1$ and $c_2$ denote unknown constants, $i=\sqrt{-1}$. Since *u*(*x*, *r*) and its LT, *U*(*s*, *r*), must be bounded at $r=0$, the value of $c_2$ must be zero since $Y_0\left(i\sqrt{ \frac{s+A_1}{A_0} }r\right)\rightarrow \infty$ as $r\rightarrow 0$. Thus, we can write

10 $$\begin{aligned} U(s,r)=c_1J_0\left(i\sqrt{ \frac{s+A_1}{A_0} }r\right) +\frac{1}{s+A_1}, \end{aligned}$$

and

11 $$\begin{aligned} \frac{d U(s,r)}{d r}=-c_1 i\sqrt{ \frac{s+A_1}{A_0}} J_1\left(i\sqrt{ \frac{s+A_1}{A_0} }r\right), \end{aligned}$$

where $J_0'(\lambda r)=-\lambda J_1(\lambda r)$. From Eqs. (10) and (11), we obtain

12 $$\begin{aligned} \frac{d U(s,r)}{d r}+A_2U(s,r)=c_1\left[ A_2J_0\left(i\sqrt{ \frac{s+A_1}{A_0} }r\right) - i\sqrt{ \frac{s+A_1}{A_0}} J_1\left(i\sqrt{ \frac{s+A_1}{A_0} }r\right) \right] + \frac{A_2}{\left(s+A_1\right)}. \end{aligned}$$

Applying LT on (4) yields

13 $$\begin{aligned} \left[\frac{d U(s,r)}{d r}+A_2U(s,r) \right]_{r=1}=0. \end{aligned}$$

From Eqs. (12) and (13), we obtain

14 $$\begin{aligned} c_1=-\frac{A_2}{\left(s+A_1\right)\left[ A_2J_0\left( i\sqrt{ \frac{s+A_1}{A_0} } \right) - i\sqrt{ \frac{s+A_1}{A_0}} J_1\left(i\sqrt{ \frac{s+A_1}{A_0} }\right) \right]}. \end{aligned}$$

Substituting (14) into (10) leads to

15 $$\begin{aligned} U(s,r)= -\frac{A_2 J_0\left(i\sqrt{ \frac{s+A_1}{A_0} }r\right) }{\left(s+A_1\right)\left[ A_2J_0\left( i\sqrt{ \frac{s+A_1}{A_0} } \right) - i\sqrt{ \frac{s+A_1}{A_0}} J_1\left(i\sqrt{ \frac{s+A_1}{A_0} }\right) \right]} +\frac{1}{s+A_1}. \end{aligned}$$

However, Eq. (15) can be written as

16 $$\begin{aligned} U(s,r)= -A_2 F(s,r)+\frac{1}{s+A_1}, \end{aligned}$$

where

17 $$\begin{aligned} F(s,r)=\frac{ J_0\left(i\sqrt{ \frac{s+A_1}{A_0} }r\right) }{\left(s+A_1\right)\left[ A_2J_0\left( i\sqrt{ \frac{s+A_1}{A_0} } \right) - i\sqrt{ \frac{s+A_1}{A_0}} J_1\left(i\sqrt{ \frac{s+A_1}{A_0} }\right) \right]}. \end{aligned}$$

Applying the inverse LT on Eq. (16), yields

18 $$\begin{aligned} L^{-1}\left( U(s,r) \right)= -A_2 L^{-1}\left( F(s,r) \right)+L^{-1}\left( \frac{1}{s+A_1} \right), \end{aligned}$$

or

19 $$\begin{aligned} u(x,r)=-A_2 f(x,r)+e^{-A_1x}, \end{aligned}$$

where *f*(*x*, *r*) is the inverse LT of *F*(*s*, *r*) so that

20 $$\begin{aligned} f(x,r)=L^{-1}\left( \frac{ J_0\left(i\sqrt{ \frac{s+A_1}{A_0} }r\right) }{\left(s+A_1\right)\left[ A_2J_0\left( i\sqrt{ \frac{s+A_1}{A_0} } \right) - i\sqrt{ \frac{s+A_1}{A_0}} J_1\left(i\sqrt{ \frac{s+A_1}{A_0} }\right) \right]} \right). \end{aligned}$$

## Analysis and exact solution of the chlorine decay model

### Analysis

The below theorem introduces the method of residues when applied to calculating the inverse LT.

#### Theorem 1

(Method of residues^31^) * Let* $s_i$ *are the poles of F*(*s*, *r*), *then* *f*(*x*, *r*) *(inverse LT of F*(*s*, *r*)) *is* $f(x,r)=\sum _{i=1}^n \text {Res}\left(e^{s_ix}F(s_i,r)\right)$ *at all poles* $s_i$.

It will be shown later that the inverse LT of the function *F*(*s*, *r*), defined in (17), using the residues, can be obtained in terms of Bessel functions with the help of their properties. In this regard, the Bessel functions $J_0(y)$, $J_1(y)$ and $J_2(y)$ are defined by the expressions:

21 $$\begin{aligned}&J_0(y)=\sum _{k=0}^{\infty } \frac{(-1)^k}{\left(k! \right)^2} \left( \frac{y}{2} \right)^{2k}, \end{aligned}$$

22 $$\begin{aligned}&J_1(y)=\sum _{k=0}^{\infty } \frac{(-1)^{k}}{ k!(k+1)! } \left( \frac{y}{2} \right)^{2k+1}, \end{aligned}$$

23 $$\begin{aligned}&J_2(y)=\sum _{k=0}^{\infty } \frac{(-1)^{k}}{ k!(k+2)! } \left( \frac{y}{2} \right)^{2k+2}, \end{aligned}$$

that satisfy the following properties:

24 $$\begin{aligned}&\frac{d}{d y} \left( J_0(\lambda y) \right)=-\lambda J_1\left(\lambda y \right) \end{aligned}$$

25 $$\begin{aligned}&\frac{d}{d y} \left( J_1(\lambda y) \right)=\frac{\lambda }{2}\left( J_0(\lambda y)-J_2(\lambda y) \right), \end{aligned}$$

26 $$\begin{aligned}&yJ_2(y)+yJ_0(y)=2J_1(y). \end{aligned}$$

### Exact solution of the chlorine decay model

The main challenge of this paper is to obtain the inverse LT of the expression in Eq. (20). The expression (20) is really complex due to the nature of the boundary conditions (2–4). This is because the denominator in expression (20) involves Bessel functions of first and second kind which leads to actual difficulties when deriving the inverse LT of the expression in Eq. (20). However, such difficulties are overcome through applying the method of residues as indicated below.

At first sight, the expression $\left(s+A_1\right)\left[ A_2J_0\left( i\sqrt{ \frac{s+A_1}{A_0} } \right) - i\sqrt{ \frac{s+A_1}{A_0}} J_1\left(i\sqrt{ \frac{s+A_1}{A_0} }\right) \right]$ has simple zeros at $s=-A_1$ and $i\sqrt{ \frac{s+A_1}{A_0} }=\lambda _1,\lambda _2,\dots \lambda _n,\dots$, and thus we find simple poles at $s_1=-A_1$ and $s_2=-A_1-A_0\lambda ^2_n,~n=1,2,3,\dots$. Therefore, the inverse LT of *F*(*s*, *r*), i.e., *f*(*x*, *r*) can be obtained from Theorem 1 by calculating the residues (Res) of $e^{sx}F(s,r)$ at $s_1=-A_1$ and $s_2=-A_1-A_0\lambda ^2_n$, and then by taking their sum. At $s_1=-A_1$, we have

27 $$\begin{aligned} \left( \text {Res}\right) _{s_1}= & {} \lim _{s\rightarrow s_1}\left( s-s_1\right) e^{sx}F(s,r),\nonumber \\= & {} e^{-A_1x}\lim _{s\rightarrow -A_1}\left( \frac{ J_0\left( i\sqrt{ \frac{s+A_1}{A_0} }r\right) }{\left[ A_2J_0\left( i\sqrt{ \frac{s+A_1}{A_0} } \right) - i\sqrt{ \frac{s+A_1}{A_0}} J_1\left( i\sqrt{ \frac{s+A_1}{A_0} }\right) \right] } \right) ,\nonumber \\= & {} e^{-A_1x} \left( \frac{ J_0\left( 0\right) }{\left[ A_2J_0\left( 0 \right) - 0 \right] } \right) ,\nonumber \\= & {} \frac{ e^{-A_1x} }{ A_2 },\quad \text {where}\quad J_0(0)=1. \end{aligned}$$

At $s_2=-A_1-A_0\lambda ^2_n$, we have

28 $$\begin{aligned} \left( \text {Res}\right) _{s_2}= & {} \lim _{s\rightarrow s_2}\left( \frac{ \left( s-s_2\right) e^{sx} J_0\left( i\sqrt{ \frac{s+A_1}{A_0} }r\right) }{\left( s+A_1\right) \left[ A_2J_0\left( i\sqrt{ \frac{s+A_1}{A_0} } \right) - i\sqrt{ \frac{s+A_1}{A_0}} J_1\left( i\sqrt{ \frac{s+A_1}{A_0} }\right) \right] } \right) ,\nonumber \\= & {} \lim _{s\rightarrow s_2}\left( \frac{ \left( s+A_1+A_0\lambda ^2_n\right) }{\left[ A_2J_0\left( i\sqrt{ \frac{s+A_1}{A_0} } \right) - i\sqrt{ \frac{s+A_1}{A_0}} J_1\left( i\sqrt{ \frac{s+A_1}{A_0} }\right) \right] } \right) \times \nonumber \\&\quad \lim _{s\rightarrow s_2}\left( \frac{ e^{sx} J_0\left( i\sqrt{ \frac{s+A_1}{A_0} }r\right) }{ s+A_1 } \right) ,\nonumber \\= & {} \lim _{s\rightarrow s_2}\left( G(s,r) \right) \times \left( \frac{ e^{-\left( A_1+A_0\lambda ^2_n\right) x} J_0\left( -\lambda _n r\right) }{ -A_0\lambda ^2_n } \right) . \end{aligned}$$

The limit of *G*(*s*, *r*) as $s\rightarrow s_2$ can be calculated using the L’Hospital’s rule as follows

29 $$\begin{aligned} \lim _{s\rightarrow s_2}\left( G(s,r) \right)= & {} \frac{ \lim _{s\rightarrow s_2} \left( s-s_2\right) }{ \lim _{s\rightarrow s_2}\left[ A_2J_0\left( i\sqrt{ \frac{s+A_1}{A_0} } \right) - i\sqrt{ \frac{s+A_1}{A_0}} J_1\left( i\sqrt{ \frac{s+A_1}{A_0} }\right) \right] } = \frac{0}{0}, \nonumber \\= & {} \frac{ \lim _{s\rightarrow s_2} ~\frac{d}{ds} \left( s+A_1+A_0\lambda ^2_n\right) }{ \lim _{s\rightarrow s_2} \frac{d}{ds}\left[ A_2J_0\left( i\sqrt{ \frac{s+A_1}{A_0} } \right) - i\sqrt{ \frac{s+A_1}{A_0}} J_1\left( i\sqrt{ \frac{s+A_1}{A_0} }\right) \right] } , \nonumber \\= & {} \frac{ 1 }{ d } , \end{aligned}$$

where

30 $$\begin{aligned} d= & {} \lim _{s\rightarrow s_2} \frac{d}{ds}\left[ A_2J_0\left( i\sqrt{ \frac{s+A_1}{A_0} } \right) - i\sqrt{ \frac{s+A_1}{A_0}} J_1\left( i\sqrt{ \frac{s+A_1}{A_0} }\right) \right] ,\nonumber \\= & {} \frac{1}{4iA_0\lambda _n} \left[ -2i(1+A_2)J_1\left( -\lambda _n \right) + i\lambda _n \left( J_0\left( -\lambda _n\right) - J_2\left( -\lambda _n\right) \right) \right] . \end{aligned}$$

Since the functions $J_0$ and $J_2$ are even and $J_1$ is odd, we obtain

31 $$\begin{aligned} d= & {} \frac{1}{4A_0\lambda _n} \left[ 2(1+A_2)J_1\left( \lambda _n \right) + \lambda _n \left( J_0\left( \lambda _n\right) - J_2\left( \lambda _n\right) \right) \right] ,\nonumber \\= & {} \frac{1}{4A_0\lambda _n} \left[ 2(1+A_2)J_1\left( \lambda _n \right) + \lambda _n J_0\left( \lambda _n\right) -\lambda _n J_2\left( \lambda _n\right) \right] ,\nonumber \\= & {} \frac{1}{4A_0\lambda _n} \left[ 2(1+A_2)J_1\left( \lambda _n \right) + \lambda _n J_0\left( \lambda _n\right) -2 J_1\left( \lambda _n\right) + \lambda _n J_0\left( \lambda _n\right) \right] ,\nonumber \\= & {} \frac{1}{2A_0\lambda _n} \left[ A_2J_1\left( \lambda _n \right) + \lambda _n J_0\left( \lambda _n\right) \right] . \end{aligned}$$

From (29) and (31), we get

32 $$\begin{aligned} \lim _{s\rightarrow s_2}\left( G(s,r) \right) = \frac{2A_0\lambda _n}{ \left[ A_2J_1\left( \lambda _n \right) + \lambda _n J_0\left( \lambda _n\right) \right] }.\end{aligned}$$

Substituting (32) into (28), yields

33 $$\begin{aligned} \left( \text {Res}\right) _{s_2}= & {} \left( \frac{ e^{-\left( A_1+A_0\lambda ^2_n\right) x} J_0\left( -\lambda _n r\right) }{ -A_0\lambda ^2_n } \right) \times \frac{2A_0\lambda _n}{ \left[ A_2J_1\left( \lambda _n \right) + \lambda _n J_0\left( \lambda _n\right) \right] },\nonumber \\= & {} -\frac{ 2e^{-\left( A_1+A_0\lambda ^2_n\right) x} J_0\left( \lambda _n r\right) }{ \lambda _n\left[ A_2J_1\left( \lambda _n \right) + \lambda _n J_0\left( \lambda _n\right) \right] }. \end{aligned}$$

Hence,

34 $$\begin{aligned} f(x,r)= \frac{e^{-A_1x}}{A_2}-2\sum _{n=1}^{\infty } \frac{ e^{-\left( A_1+A_0\lambda ^2_n\right) x} J_0\left( \lambda _n r\right) }{ \lambda _n\left[ A_2J_1\left( \lambda _n \right) + \lambda _n J_0\left( \lambda _n\right) \right] }. \end{aligned}$$

Inserting (34) into (19) leads to

35 $$\begin{aligned} u(x,r)=2\sum _{n=1}^{\infty } \frac{ A_2J_0\left( \lambda _n r\right) e^{-\left( A_1+A_0\lambda ^2_n\right) x} }{ \lambda _n\left[ A_2J_1\left( \lambda _n \right) + \lambda _n J_0\left( \lambda _n\right) \right] } , \end{aligned}$$

where the symbols $\lambda _n$ denote the roots of the equation:

36 $$\begin{aligned} A_2J_0\left( \lambda _n \right) - \lambda _n J_1\left( \lambda _n\right) =0. \end{aligned}$$

Using (36), the solution (35) can be written as

37 $$\begin{aligned} u(x,r)=2\sum _{n=1}^{\infty } \frac{ \lambda _n J_1\left( \lambda _n \right) J_0\left( \lambda _n r\right) }{ \left( A^2_2+ \lambda ^2_n \right) J^2_0\left( \lambda _n\right) } e^{-\left( A_1+A_0\lambda ^2_n\right) x}, \end{aligned}$$

which agrees with solution in Ref.^4^ derived by the separation of variables technique.

## Discussion of results

The dimensionless cup-mixing average concentration is defined by

38 $$\begin{aligned} u_{\text {av}}=2\int _{0}^{1} u(x,r)~rdr. \end{aligned}$$

Substituting (37) into (38), yields

39 $$\begin{aligned} u_{\text {av}}=4\sum _{n=1}^{\infty } \frac{ \lambda _n J_1\left( \lambda _n \right) }{ \left( A^2_2+ \lambda ^2_n \right) J^2_0\left(\lambda _n\right) } e^{-\left(A_1+A_0\lambda ^2_n\right)x} \int _{0}^{1} rJ_0\left(\lambda _n r\right)dr, \end{aligned}$$

or

40 $$\begin{aligned} u_{\text {av}}=4\sum _{n=1}^{\infty } \frac{ J^2_1\left( \lambda _n \right) }{ \left( A^2_2+ \lambda ^2_n \right) J^2_0\left(\lambda _n\right) } e^{-\left(A_1+A_0\lambda ^2_n\right)x}. \end{aligned}$$

After including the relation (36) we obtain

41 $$\begin{aligned} u_{\text {av}}=4\sum _{n=1}^{\infty } \frac{ A^2_2 }{ \lambda ^2_n\left( A^2_2+ \lambda ^2_n \right) } e^{-\left(A_1+A_0\lambda ^2_n\right)x}. \end{aligned}$$

If $$A_2\rightarrow \infty $$, then

42 $$\begin{aligned} u_{\text {av}}=4\lim _{A_2\rightarrow \infty }\left(\sum _{n=1}^{\infty } \frac{ A^2_2 }{ \lambda ^2_n\left( A^2_2+ \lambda ^2_n \right) } e^{-\left(A_1+A_0\lambda ^2_n\right)x} \right), \end{aligned}$$

which gives

43 $$\begin{aligned} u_{\text {av}}=\sum _{n=1}^{\infty } \frac{ 4 }{ \lambda ^2_n } e^{-\left(A_1+A_0\lambda ^2_n\right)x}. \end{aligned}$$

Moreover, if $A_2\rightarrow 0$ then Eq. (19) implies

44 $$\begin{aligned} u(x,r)= e^{-A_1x}, \end{aligned}$$

and the corresponding $u_{\text {av}}$ is obtained from Eq. (38) as

45 $$\begin{aligned} u_{\text {av}}=2\int _{0}^{1} e^{-A_1x}~rdr=e^{-A_1x}. \end{aligned}$$

Therefore, Eq. (41) gives the general expression for the $u_{\text {av}}$ while Eqs. (43) and (45) are limiting cases. According to Biswas et al.^4^, three roots $\lambda _1$, $\lambda _2$, and $\lambda _3$ of Eq. (36) were used. In addition, the following fitting functions were used to reproduce ($\lambda _1$, $\lambda _2$, $\lambda _3$) in terms of $A_2$ at several ranges.

46 $$\begin{aligned}&\text {(i) For} ~ 0.01\le A_2<1,\nonumber \\&\lambda _1=1.29861 \left(A_2\right)^{0.477433},~ \lambda _2=4.00946 \left(A_2\right)^{0.0119894} ,~ \lambda _3=7.11555 \left(A_2\right)^{0.00376107}, \end{aligned}$$

47 $$\begin{aligned}&\text {(ii) For} ~ 1\le A_2<10,\nonumber \\&\lambda _1=1.30427 \left(A_2\right)^{0.239289},~ \lambda _2=4.05693 \left(A_2\right)^{0.0927629} ,~ \lambda _3=7.10846 \left(A_2\right)^{0.0463785}, \end{aligned}$$

48 $$\begin{aligned}&\text {(iii) For} ~ 10\le A_2<1000,\nonumber \\&\lambda _1=2.10218 \left(A_2\right)^{0.021361},~ \lambda _2=4.86441 \left(A_2\right)^{0.0200514} ,~ \lambda _3=7.71165 \left(A_2\right)^{0.0182292}. \end{aligned}$$

In order to have a numerical comparison between the current calculations of the first three roots $\lambda _1$, $\lambda _2$, and $\lambda _3$ of Eq. (36) and the corresponding results in^4^ (using Eqs. (46)–(48)), we may write Eq. (36) as a function of $\lambda$ in the form:

49 $$\begin{aligned} f(\lambda )=A_2J_0\left( \lambda \right) - \lambda J_1\left(\lambda \right).\end{aligned}$$

**Figure 1:**
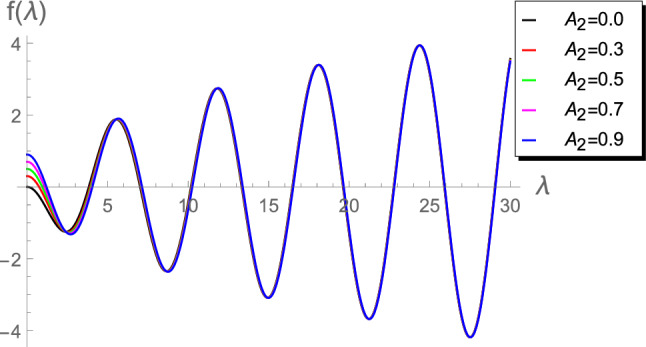
Plot of $f(\lambda )$ vs $\lambda$ for $A_ 2 =\left\lbrace 0.0, 0.3, 0.5, 0.7, 0.9\right\rbrace$.

**Figure 2:**
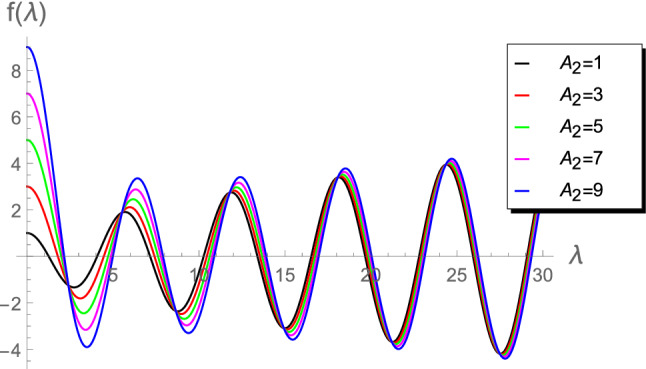
Plot of $f(\lambda )$ vs $\lambda$ for $A_2 =\left\lbrace 1, 3, 5, 7, 9 \right\rbrace$.

**Figure 3:**
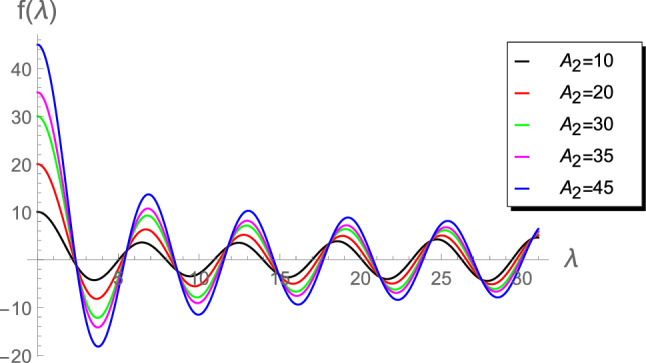
Plot of $f(\lambda )$ vs $\lambda$ for $A_2= \left\lbrace 10, 20, 30, 35, 45\right\rbrace$.

**Figure 4:**
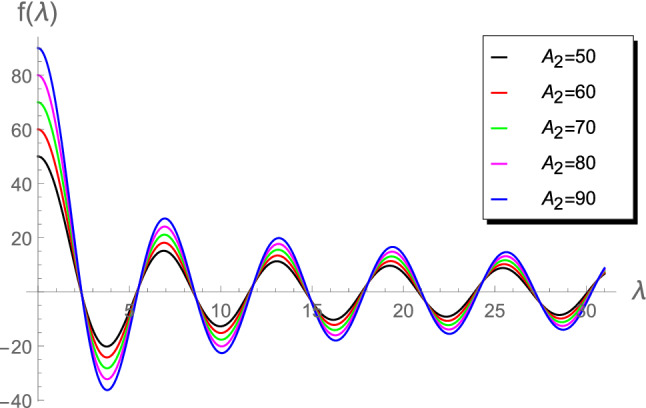
Plot of $f(\lambda )$ vs $\lambda$ for $A_2= \left\lbrace 50, 60, 70, 80, 90 \right\rbrace$.

**Figure 5:**
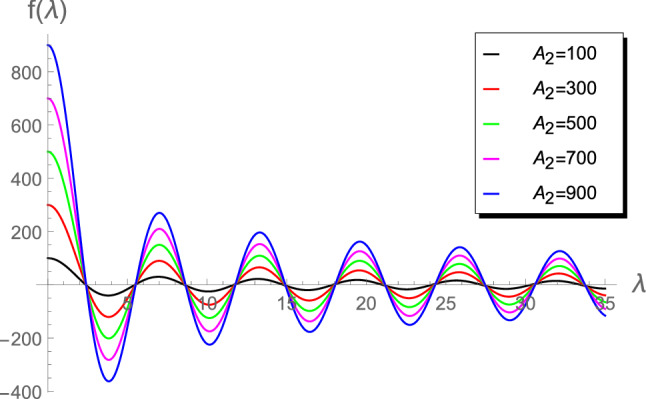
Plot of $f(\lambda )$ vs $\lambda$ for $A_2 = \left\lbrace 100, 300, 500, 700, 900 \right\rbrace$.

Figures 1, 2, 3, 4 and 5) highlight details about the roots of $f(\lambda )$ for different values of $A_2$. We verify that we have an infinite number of roots. In addition, all the roots of $f(\lambda )$, excepting the first one, are nearly identical for small $A_2\in (0.01,1]$, as shown by Fig. 1. However, for the range $1\le A_2<10$, Fig. 2 reveals that the first seven roots are considerably different, while the others have approximately the same values. Figure 3 indicates that the first two roots are nearly identical, while the rest of roots are considerably different for the range $10\le A_2<45$. For higher values of $A_2$, namely in the ranges $50\le A_2<90$ and $100\le A_2<900$, Figs. 4 and 5 reveal that the roots of $f(\lambda )$ are nearly identical. However, the results introduced in^4^ were mainly depend on obtaining the first three roots $\lambda _1$, $\lambda _2$ and $\lambda _3$ and, therefore, the proposed approach is more accurate.

Table 1 presents the three roots $\lambda _1$, $\lambda _2$ and $\lambda _3$ of Eq. (36) and the corresponding results from^4^ (using Eq. (46)) in the interval $A_2\in (0.01,1]$. The calculations of the present roots are accomplished through MATHEMATICA. The absolute errors listed in Table 1 show that the results presented in^4^ agree with the obtained ones only up to two/three digits at most. This means that the new approach leads to better results than those of^4^ after 3 decimal places. A similar conclusion is also obtained in Table 2 regarding $\lambda _1$, $\lambda _2$ and $\lambda _3$ for the range $1\le A_2<10$.

**Table 1:** Comparisons of present roots $\lambda _1$, $\lambda _2$, and $\lambda _3$ of Eq. (36) and the corresponding results in Ref.^4^ using Eqs. (46) ($0.01\le A_2<1$).

$A_2$	Present			Ref.^4^			Absolute error		
	$\lambda _1$	$\lambda _2$	$\lambda _3$	$\lambda _1$	$\lambda _2$	$\lambda _3$	$\lambda _1$	$\lambda _2$	$\lambda _3$
0.01	0.141245	3.83431	7.01701	0.144083	3.79409	6.99337	0.002838	0.040230	0.023645
0.10	0.441682	3.85771	7.02983	0.432559	3.90029	7.05419	0.009122	0.042576	0.024369
0.20	0.616975	3.88351	7.04403	0.602237	3.93283	7.07261	0.014738	0.049329	0.028579
0.50	0.940771	3.95937	7.08638	0.932732	3.97628	7.09702	0.008038	0.016907	0.010643

**Table 2:** Comparisons of present roots $\lambda _1$, $\lambda _2$, and $\lambda _3$ of Eq. (36) and the corresponding results in Ref.^4^ using Eqs. (47) ($1\le A_2<10$).

$A_2$	Present			Ref.^4^			Absolute error		
	$\lambda _1$	$\lambda _2$	$\lambda _3$	$\lambda _1$	$\lambda _2$	$\lambda _3$	$\lambda _1$	$\lambda _2$	$\lambda _3$
1	1.25578	4.07948	7.15580	1.30427	4.05693	7.10846	0.04849	0.02255	0.04734
2	1.59945	4.29096	7.28839	1.53957	4.32635	7.34069	0.05987	0.03539	0.05230
5	1.98981	4.71314	7.61771	1.91701	4.71016	7.65936	0.07281	0.00298	0.04166

**Table 3:** Comparisons of present roots $\lambda _1$, $\lambda _2$, and $\lambda _3$ of Eq. (36) and the corresponding results in Ref.^4^ using Eqs. (48) ($10\le A_2<1000$).

$A_2$	Present			Ref.^4^			Absolute error		
	$\lambda _1$	$\lambda _2$	$\lambda _3$	$\lambda _1$	$\lambda _2$	$\lambda _3$	$\lambda _1$	$\lambda _2$	$\lambda _3$
10	2.17950	5.03321	7.95688	2.20816	5.09427	8.04223	0.02867	0.06105	0.08535
50	2.35724	5.41120	8.48399	2.28540	5.26135	8.28167	0.07185	0.14985	0.20231
100	2.38090	5.46521	8.56783	2.31949	5.33498	8.38698	0.06141	0.13023	0.18085

**Figure 6:**
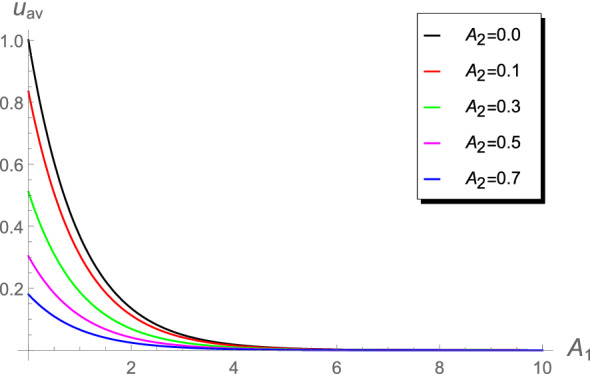
The $u_{\text {av}}$ against $A_1$ at different values of $A_2$, $A_0$= 1.4.

**Figure 7:**
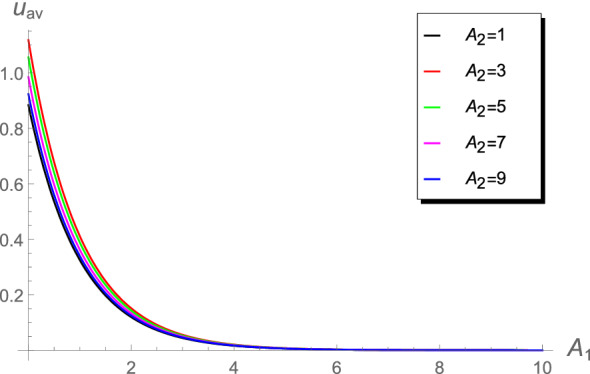
The $u_{\text {av}}$ against $A_1$ at different values of $A_2$, $A_0=1.4\times 10^{-3}$.

**Figure 8:**
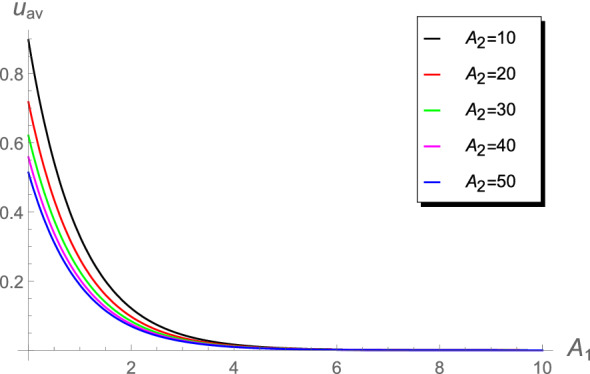
The $u_{\text {av}}$ against $A_1$ at different values of $A_2$, $A_0=1.4\times 10^{-2}$.

**Figure 9:**
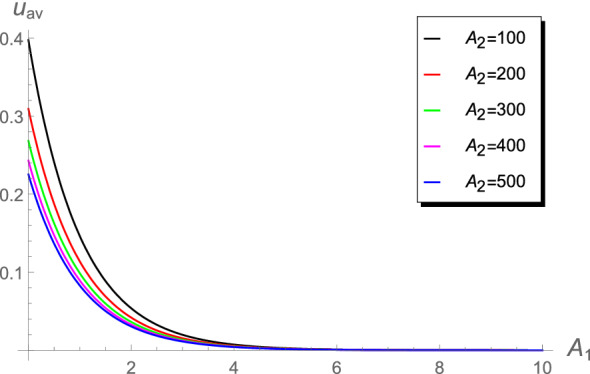
The $u_{\text {av}}$ against $A_1$ at higher values of $A_2$, $A_0=1.4\times 10^{-2}$.

Table 3 shows that the absolute errors increase in the range $10\le A_2<1000$. Such differences in the values may lead to differences when calculating the chlorine concentration or the cup-mixing average concentration. The behavior of the cup-mixing average concentration $u_{\text {av}}$, at the outlet $x=1$ of a pipe, versus $A_1$ are displayed in Figs. 6, 7, 8 and 9 for several values of $A_0$ and $A_2$. These figures indicate that the $u_{\text {av}}$ is always a decreasing function in the parameter $A_1$. This means that the cup-mixing average concentration decays with increasing the parameter $A_1$. In conclusion, the proposed approach gives a clear and precise solution of the mathematical model.

## Conclusion

The two-dimensional chlorine-model was theoretically solved. The exact solution was obtained by applying the LT. The method of residues was adopted to obtain the inverse LT of complex expressions and the solution was expressed in terms of Bessel functions of the first and the second kinds of order zero. The exact solutions agree with those published previously using the method of separation of variables. However, the obtained numerical results are superior to than those reported in^4^ due to the limitations on the calculation of the roots of Eq. (36). Previous studies conducted the numerical calculations based on various fitting curves to predict the values of such roots at prescribed values of $A_2$. Such fitting scheme involves some numerical errors as verified in Tables (1-3), where the absolute errors may be relevant for many cases. The expression of the dimensionless cup-mixing average concentration was analytically derived. The results proved that the new approach gives reliable and accurate solutions of the problem.
